# CORSA study finds spike-specific blunted immune responses in lymphoma patients after SARS-CoV-2 vaccine

**DOI:** 10.3389/fimmu.2026.1756325

**Published:** 2026-05-20

**Authors:** Lucia Mazzotti, Gerardo Musuraca, Fabio Affaticati, Esther Bartholomeus, Diana Campillo-Davo, Carole Faghel, Chiara Zingaretti, Flavia Foca, Claudio Cerchione, Valentina Ancarani, Patricia Borges de Souza, Fabio Nicolini, Anna De Lucia, Anna Gaimari, Oriana Nanni, Vittorio Sambri, Giovanni Martinelli, Francesco Malaspina, Pieter Meysman, Eva Lion, Massimiliano Mazza

**Affiliations:** 1 IRCCS Istituto Romagnolo per lo Studio dei Tumori (IRST) “Dino Amadori”, Meldola, Italy; 2 ADREM Data Lab of the Mathematics and Computer Science Department, University of Antwerp, Antwerp, Belgium; 3 Laboratory of Experimental Hematology (LEH), Vaccine and Infectious Disease Institute (VAXINFECTIO), Faculty of Medicine and Health Sciences, University of Antwerp, Antwerp, Belgium; 4 Division of Hematology, Antwerp University Hospital (UZA), Edegem, Belgium; 5 Department of Medical and Surgical Sciences, Alma Mater Studiorum, University of Bologna, Bologna, Italy; 6 Unit of Microbiology, Greater Romagna Area Hub Laboratory, Cesena, Italy; 7 Department of Hematology and Sciences Oncology, Institute of Haematology “L. and A. Seràgnoli” S. Orsola, University Hospital in Bologna, Bologna, Italy

**Keywords:** immunotherapy, lymphoma, rituximab, SARS-CoV-2, TCR sequencing, COVID-19

## Abstract

**Introduction:**

Hematological patients are at higher risk of severe SARS-CoV-2 infection and exhibit impaired vaccine responses due to disease and therapy. Treatments like Rituximab are known to compromise immune function, reducing the generation of protective antibodies.

**Methods:**

In the CORSA trial, we evaluated humoral and cellular responses to messenger RNA vaccines against SARS-CoV-2 in 77 patients with hematological malignancies receiving active treatment and in matched healthy controls. Peripheral blood samples were processed to assess Spike-specific immunoglobulin G titers, T-cell responses by enzyme-linked immunospot assay, and T-cell receptor repertoire sequencing from baseline to six months after vaccination to determine clonal breadth and depth of Spike-specific T-cell receptor clones.

**Results:**

Seroconversion occurred in only 8% and 21% of patients after the first and second dose, compared to 93% and 100% of healthy controls. Despite poor antibody responses, 69% of seronegative patients showed measurable T-cell activity, suggesting some level of vaccine-induced protection. Spike-specific TCR repertoire analysis in lymphoma patients (LP) and HC revealed significantly broader clonal breadth (p = 0.0013) and higher clonal depth (p = 0.0265) in HC at day 50 versus baseline, while blunted diversification in LP (p = 0.0407).

**Conclusions:**

Lymphoma patients showed significantly weaker Spike-specific clonal responses and reduced diversity compared with healthy controls, supporting the association with increased patient vulnerability.

## Graphical Abstract

**Figure:**
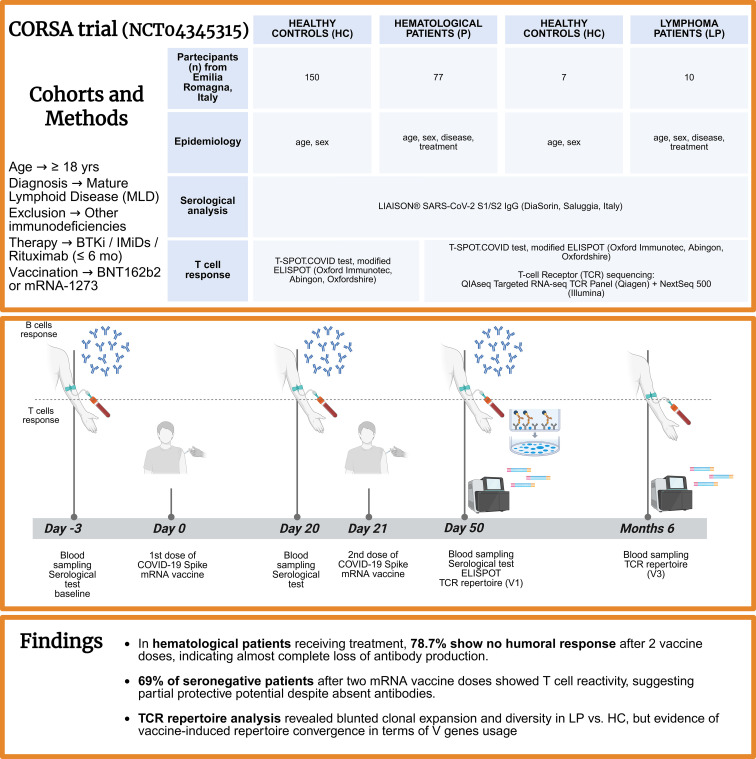

## Introduction

Severe Acute Respiratory Syndrome Coronavirus 2 (SARS-CoV-2), the etiological agent of coronavirus disease 2019 (COVID-19), led in 2020 to a global health crisis with profound consequences, while SARS-CoV-2, influenza, and respiratory syncytial virus (RSV) circulation during the 2024–2025 respiratory season led to a “tridemic” with a severe impact on the health system of many countries (1, 2).

Children and immunocompromised individuals, including patients with oncological diseases, congenital immunodeficiencies, or those who have undergone solid organ or hematopoietic stem cell transplants, face a heightened risk of severe respiratory viral infections, which often lead to serious complications, including increased morbidity and mortality, prolonged hospitalizations, respiratory failure, and reduced efficacy of initial antibiotic treatments (3–5). Among those, hematological malignancies (HM) have a significantly worse COVID-19-related prognosis (6–8), particularly those treated with drugs affecting the B lymphocyte population, which result in reduced humoral immune functions. Several studies have already shown that hematological patients undergoing treatment do not always seroconvert after being infected by SARS-CoV-2 and have longer viral clearance times (6, 9), in accordance with a less effective immune system. However, antibody testing alone is insufficient to estimate the protection induced against SARS-CoV-2 infection since it does not consider the antigen-specific T cell activity exerted on infected cells or in support of B cells in the restraining of viral spread. Studies have demonstrated that mRNA (BNT162b2, mRNA-1273) and viral vector COVID-19 vaccines elicit strong T cell responses (10) and that, while SARS-CoV-2 variants could escape neutralizing antibodies, the cell-mediated immune response is very resilient and stays effective (11, 12).

Emerging data suggest that lymphoma patients face worse outcomes when infected with SARS-CoV-2, including higher rates of hospitalization, severe disease, and a mortality rate of around 30% for hospitalized patients, with patient-related factors like age, active disease, and comorbidities worsening the prognosis (13–15). Lymphoma encompasses a heterogeneous group of hematological malignancies characterized by the uncontrolled proliferation of lymphocytes. Clinical and immunological features such as hypogammaglobulinemia, neutropenia, and depletion of B and T cells are frequently observed in affected patients, although their occurrence and extent vary according to disease subtype, stage and, most prominently, prior to or during immunochemotherapy. The disease can be broadly categorized into Hodgkin lymphoma (HL) and non-Hodgkin lymphoma (NHL), with the latter including aggressive and indolent subtypes such as diffuse large B cell lymphoma (DLBCL) and follicular lymphoma (FL). The immunosuppressive nature of lymphoma, particularly in B cell lymphomas, is exacerbated by treatments targeting the B cell compartment, such as anti-CD20 monoclonal antibodies, such as rituximab, which lead to wide and prolonged B cell depletion (16–18). The prognosis varies among lymphoma subtypes as, for example, DLBCL patients tend to have worse outcomes compared to those with indolent lymphomas like follicular lymphoma (15). The impaired ability in these patients to mount effective humoral and cellular immune responses further complicated their disease management during the pandemic and still poses an issue in the advent of similar situations. Therefore, it is crucial to understand immune dynamics in these diseases, especially after vaccination and in particular for lymphoma, and how severe immune impairment may affect viral infection clearance. This will in turn improve patient outcomes and can guide treatment strategies in this type of high-risk population, considering the pandemic transitions into an endemic state and the possibility of new epidemics.

The CORSA trial (NCT04345315) was a prospective multicentric research study from the Istituto Romagnolo per lo Studio dei Tumori “Dino Amadori” – IRST to investigate the immunologic response to mRNA SARS-CoV-2 vaccines in patients with HM and healthy individuals as controls (HC). All enrolled subjects signed an informed consent according to the Helsinki Declaration (19) and underwent vaccination through and according to the Italian national vaccination campaign. Specifically, both cohorts received two doses of mRNA-based vaccine (BTN162b2, also known as Comirnaty, with a 21-day interval, or mRNA1273, also known as Spikevax, day 0 and day 28). Immunological responses were monitored for the following six months.

We first evaluated the humoral and cellular response to the anti-SARS-CoV-2 mRNA vaccines BTN162b2 and mRNA1273 in all patients with mature B cell malignancies undergoing treatment with rituximab alone or in combination with Bruton’s tyrosine kinase inhibitors (BTKi) or immunomodulatory drugs (IMiDs). These drugs preferentially target lymphocytes and can severely impact immune responses to vaccination (20–23). In a subsequent analysis, we focused on a cohort of lymphoma patients (LP) treated with the aforementioned regimens and investigated specific SARS-CoV-2-associated T cell responses by analyzing T cell receptor (TCR) repertoires and the correlation between serological and cellular responses between LP and HC individuals. Since TCR repertoires denote a unique T cell clonotype signature in an individual, and hence T cell antigen specificity (24), we studied the SARS-CoV-2-specific T cell response in terms of expansion (clonal depth) and diversification (clonal breadth) of T lymphocyte clonotypes in response to vaccination over time by Next Generation Sequencing (NGS), characterizing the CDR3 sequences of the TCR β-chains from peripheral blood samples. This in-depth TCR repertoire analysis goes beyond previous reports, where mostly interferon gamma (IFN-γ) production was evaluated to describe T cell responses in hematological patients (25–27). While IFN-γ production offers functional insights, TCR sequencing uniquely resolves the clonal architecture and dynamics of antigen-specific responses, providing a molecular blueprint of immunological memory.

## Materials and methods

### Population

The CORSA trial (NCT04345315) eligibility criteria were: age ≥ 18 years; confirmed diagnosis of mature lymphoid disease (MLD); no immunodeficiency other than the one caused by MLD or its therapy; undergoing therapy with either a BTKi, IMiDs or rituximab, alone or in combination for no longer than 6 months before vaccination; candidate to receive SARS-CoV-2 vaccination (either with BNT162b2 or mRNA-1273) according to the Italian guidelines. Patients undergoing rituximab maintenance were evaluated case by case to determine whether treatment could be suspended to grant a therapy-free vaccine administration window. Medical history, including disease stage, previous and ongoing treatments for hematological diseases, and COVID-19 history, was collected for all the participants at enrollment. The clinical diaries of patients who presented an immune response were analyzed to search for possible modifying variables, such as other immunosuppressant drug usage, previous influenza vaccination, presence of autoimmune diseases, and allergy status. A subgroup of the HC subjects was selected based on age and sex.

Our study examined male and female humans, and similar findings are reported for both sexes.

### Antibody response evaluation

All consenting patients and HC underwent blood sample collection within three days before first vaccine injection to assess the pre-exposure value of anti-SARS-CoV-2 antibodies and after 20 (+/- 7) and 50 (+/- 7) days from first vaccine administration to evaluate the amount of antibodies elicited. Anti-SARS-CoV-2 Spike IgG serum levels were determined by an indirect chemiluminescence immunoassay (CLIA) for the fully automated quantitative determination of anti-S1 and anti-S2 IgG (LIAISON^®^ SARS-CoV-2 S1/S2 IgG, DiaSorin, Saluggia, Italy) on a LIAISON^®^ XL Analyzer. In this assay, anti-SARS-CoV-2 S1/S2 IgG concentrations are expressed as Arbitrary Units/mL (AU/mL), with sensitivity ranging 3.8–400 AU/mL. Samples were considered seropositive if equal or above 15.0 AU/mL, equivocal if between 15 AU/mL and 12 AU/mL, and seronegative below 12 AU/mL. Concentrations greater than the upper limit were expressed as > 400 AU/mL.

### T cell response evaluation through interferon-gamma release

T cell response was evaluated after 50 (+/- 7) days from first vaccine administration. Cell-mediated immune response to SARS-CoV-2 was assessed by a non-automated test (T-SPOT.COVID Test, Oxford Immunotec, Abingdon, Oxfordshire) based on a modified Enzyme-Linked ImmunoSpot (ELISPOT) technology (28–30). This test can detect the presence of T cells sensitized to SARS-CoV-2 in a whole blood sample and is designed to activate both CD4 and CD8 effector cells for their capacity to produce IFN-γ when stimulated by specific antigens. To perform the test, peripheral blood mononuclear cells (PBMCs) isolated from whole blood samples were exposed to two separate panels of SARS-CoV-2 antigens derived from S (Panel A) and N (Panel B) proteins, to the non-specific mitogenic stimulator phytohemagglutinin (PHA) (positive control) and were incubated also without any antigen (Nil) (negative control/regular background reactivity of the sample if its count is ≤ 10 spots). The test was considered reactive if (Panel A - Nil) and/or (Panel B - Nil) ≥ 8 spots, indicating that the sample contained effector T cells sensitized to SARS-CoV-2. The upper limit for Panel A and Panel B was set at 20 spots. The test result was non-reactive if both (Panel A - Nil) and (Panel B - Nil) ≤ 4 spots. Results where (Panel A - Nil) or (Panel B - Nil) > 4 spots but < 8 spots were considered equivocal and excluded from the analysis.

### Statistical analysis

Categorical data were expressed as absolute and percentage frequencies, while continuous variables were expressed as median and range or interquartile range (IQR). The association between demographics, clinical factors, and antibody values was assessed by nonparametric tests: chi-square or Fisher’s exact test for categorical variables and Wilcoxon rank sum test for continuous variables. The McNemar test was used to determine whether there were significant differences in antibody titers and IFN-γ spot formation against the S protein and the N protein. Patients treated with rituximab were stratified according to the time interval between treatment discontinuation and the first vaccine dose into four groups: ongoing treatment, ≤3 months, 3–12 months, and >12 months. For patients receiving ongoing therapy, the end of treatment was defined as the date of first vaccine administration. Antibody titers at each timepoint were compared across groups using the Mann–Whitney U test for pairwise comparisons (ongoing vs each group), given the non-normal distribution of the data. Results were reported as two-sided p-values, and statistical significance was defined as p < 0.05. All statistical analyses were performed using Stata/SE version 15.1 for Windows (StataCorpLP, College Station, TX, USA).

### Biological materials for SARS-CoV-2-associated TCR repertoire dynamics study

We considered two cohorts of individuals, 10 LP and 7 HC, randomly chosen from the CORSA study. Samples characteristics are reported in the Population paragraph. Whole blood samples were collected to study the TCR repertoire specificity against SARS-CoV-2 proteins before and after two BNT162b2 vaccination cycles at different timepoints: baseline (before vaccination), V1 (50 days after the first dose), V3 (6 months after the first dose). PBMCs from whole blood samples were isolated using Ficoll density gradient centrifugation, subsequently lysed in RLT buffer (Qiagen) and stored at -20 °C before further processing.

### RNA extraction and library preparation

Total RNA was extracted from PBMCs using the RNeasy mini kit (Qiagen). RNA quantification and quality were assessed by Qubit RNA HS Assay (Thermo Fisher) and by RNA 6000 Pico Kit for (2100 Bioanalyzer Systems), respectively. RNA Integrity Number (R.I.N.) value, together with DV600 calculation, representing the percentage of RNA fragments above 600 nucleotides, was used to qualify purified RNA. RNA was processed to generate TCR libraries, covering α, β, γ, and δ TCR chains, using the QIAseq Targeted RNA-seq Panel for T-cell Receptor (Qiagen), following the supplier’s instructions (unpaired TCR chain datasets). Each RNA sample gave rise to three independent libraries. The concentration of the libraries was measured by Qubit HS dsDNA kit (Qubit 4.0 fluorometer, Thermo Fisher), and the length distribution of the fragments was determined by Tapestation using the D1000 screen tape (Agilent).

### TCR Sequencing

The libraries were pooled isochorically to a final concentration of 4 nM, to keep the sequencing reads balanced with the sample input. The pool was further diluted to 1.1 pM and loaded to the NextSeq 500/550 Mid Output Kit v2.5 (300 Cycles, 130M reads, Illumina) on a NextSeq 500 (Illumina) for sequencing, with the following characteristics: paired-end sequencing with read 1259 cycles, read 2–39 cycles, dual barcodes of each 10 bp and 5% PhiX as internal control. Repertoire sequencing was focused on the CDR3 regions of the TCR chains. Samples were divided into 2 sequencing runs: Run 1 containing 25 samples (75 library prep reactions; 54.33 Gbp yield) and Run 2 containing 26 samples (78 library prep reactions; 47.16 Gbp yield).

### Data preprocessing

The raw TCR read files were pre-processed with MiXCR (version 3.0.7) (https://mixcr.com/), followed by analysis using a combination of Python (version 3.11.8) and R (version 4.3.3). We defined a TCR β or α clonotype as the combination of a V-segment, the CDR3 amino acid sequence, and a J-segment. Sequences generated by non-functional V and J genes were removed through a lookup search to the IMGT database (https://www.imgt.org/). Furthermore, to minimize the impact of sequencing errors and non-functional rearrangements, CDR3 sequences were filtered by discarding those shorter than three or longer than 30 amino acids. Additionally, sequences lacking a cysteine residue at the N-terminus or either a cysteine, tryptophan, or phenylalanine at the C-terminus were excluded. Samples for individuals 1 (39700 HC) and 10 (668 LP) were also excluded after assessing pre-existing seropositivity (pre-vaccination antibody titers of 60.1 and 8.7 AU/mL, respectively). Analyses focused on β-chain sequences of the TCR repertoire.

### SARS-CoV-2 specificity predictions and repertoire resampling

TCR repertoires were annotated with TCR-epitope binding predictions by way of ImmuneWatch’s DETECT algorithm (version 1.0, https://www.immunewatch.com/solutions/imw-detect). By leveraging a curated database, DETECT can summarize the specificity that a TCR amino acid sequence shows for a specific epitope in a very conservative scoring system.

We used the number of unique clonotypes present in each sample and the number of unique clonotypes associated with pathogens different from SARS-CoV-2, calculating and plotting their frequencies. The same was performed with the SARS-CoV-2-Spike specific viral ratios.

The cut-off employed to ascertain epitope specificity was the suggested 0.2, and the selected epitopes for annotation were those constituting the Spike glycoprotein of SARS-CoV-2. To assess the specificity of TCR responses toward SARS-CoV-2 Spike, we analyzed the TCR repertoire using a secondary reference database that includes TCRs associated with a variety of pathogens. To compute the clonal breadth, we employed the DETECT algorithm maintaining the score threshold of 0.2 consistently across all analyses. For other pathogens, clonal breadth was calculated based on DETECT predictions targeting non-self antigens, explicitly excluding sequences identified as human or SARS-CoV-2. The “other” category in the DETECT output (https://immunewatch.gitlab.io/detect-docs/epitope-support) includes a broad range of predicted pathogens, such as ‘Human betaherpesvirus 5’, ‘HIV’, ‘Yellow fever virus’, ‘Human gammaherpesvirus 4’, ‘Hepatitis C virus’, ‘HPV’, ‘HIV,HIV-1’, ‘Influenza A virus’, ‘Mycobacterium’, ‘Influenza B virus’, ‘Hepatitis B virus’, ‘Influenza A virus, Influenza B virus’, ‘Bacteria’, ‘Triticum aestivum’, ‘Human mastadenovirus C’, ‘Dengue Virus 1’, ‘HTLV-1’, ‘Chrysanthemum virus B’, ‘Human alphaherpesvirus 2’, ‘HIV-1,HIV’, ‘Rotavirus’, ‘Plasmodium falciparum’, ‘Pseudomonas aeruginosa’, ‘Dengue Virus 3, Dengue Virus 4’, ‘Human alphaherpesvirus 1’. Following confirmation of normal distribution, a paired t-test was conducted to compare the fold changes in the viral ratios (V1/baseline) between SARS-CoV-2-Spike and other pathogens.

Significant differences in sequencing depths led to spurious results in the initial phases of the analysis. The 7 samples for the third timepoint (V3) belonging to HC were identified to have significantly larger repertoire sizes (number of unique CDR3 sequences) and were thus downsampled.

Resampling over 1.000 permutations was performed to evaluate whether the observed abundance of predicted antigen-specific TCRβ clonotypes in the final downsample represented an extremely high value. For each HC at the V3 timepoint, resampling was performed without replacement to avoid duplicates and weighted considering clone fraction given that expanded clones are naturally more likely to be observed. To avoid library size effects, the number of unique clones sampled was maintained constant across repertoires. Sampled sequences were then compared to the reference set of predicted SARS-CoV-2 Spike-specific clonotypes based on the exact matching of the amino acid sequence and V and J genes. The overall representation of predicted antigen-specific clonotypes was quantified for each repertoire by summing the relative abundance of all matched sequences. This value was averaged across all repertoires, producing an empirical distribution of expected values of predicted antigen-specific clones under random, abundance-weighted selection. The downsampled value was then compared against this empirical distribution to assess it did not lay in the extreme tail, but rather in the 23rd percentile. Each sample was reduced to match a reference average β-chain repertoire size of 59,749 unique CDR3 sequences. Clonotypes were resampled based on their clonal fraction within the sample. After resampling, clonal fractions were recalculated to account for the newly discarded sequences in order to reduce the bias (**Supplementary Figure 1**).

For one of the lymphoma samples, the clonotype composed of sequence *CATSLANTGELFF* (V gene TRBV15 and J gene TRBJ2-2) was discarded at this step and deemed as a false positive, as it was observed to represent a very elevated ~0.02 of the clonal fraction that also did not follow the expected vaccine antigen stimulation trend.

### SARS-CoV-2 clonal depth and breadth correlation to antibody titers

In order to get a deeper understanding of the variability and depth of anti-Spike T cell responses at the clonal level after vaccination, we performed TCR sequencing in LP (n = 10) and HC (n = 7) individuals, comparing the frequency of clonotypes associated with recognizing Spike-derived peptides. This was possible since an extensive analysis of TCRs associated with SARS-CoV-2 recognition was previously reported by us and others (33). The SARS-CoV-2-specific TCR clonal depth was defined as the proportion of the total TCR repertoire made up of clones predicted by DETECT to target the virus, while clonal breadth referred to the proportion of unique clonotypes identified by DETECT as virus-specific. These two metrics were correlated with antibody titers at the second and third timepoints using Pearson’s correlation test.

### V Gene usage clustering and latent projections

For every sample, the β-chain clonal depth specific to SARS-CoV-2 was calculated at a V gene level. To assess the similarity in V gene usage profiles between samples, Pearson’s correlation coefficients were computed for all pairwise comparisons, generating a correlation matrix for each timepoint. Hierarchical clustering was applied to the correlation matrices. Clustering was performed using Euclidean distance as the distance metric and complete linkage as the agglomeration method. The pheatmap package in R was used for this purpose. The V gene-level clonal depth values were used as input for Principal Component Analysis (PCA). This dimensionality reduction enabled projection of the samples into two-dimensional space, facilitating visual comparison of repertoire profiles. Biplots were generated for each timepoint by annotating the V gene contributions to the PCA latent space.

### Linear mixed models

Linear Mixed Models (LMMs) were implemented with the R package lme4 (version 1.1-35.5). The LMM models were used to explain how the SARS-CoV-2 specific clonal depth and breadth vary based on the fixed effects of the condition (LP vs HC), the three timepoints, and their interaction while accounting for random differences between individual samples. The random effect for each individual allows each patient to have their baseline level, while still estimating the overall effect of the condition and the time. This results in the following formula:

Depth || Breadth = Condition x Timepoint + (1 | Patient).

### HLA prediction

In-silico HLA typing was performed using the hla3 tool (https://github.com/kmayerb/hla3) (31) and HLAGuessr (version 0.1.6, https://github.com/statbiophys/HLAGuessr) (32) based on the TCR β repertoires for the former, and both β and α sequences for the latter, combined across timepoints. Both tools predicted two alleles for the A, B, and C HLA loci by ranking predictions by likelihood and selecting the top two results. Alleles were considered reliable if both tools provided concordant predictions.

## Results

### Hematological patients show severe impairment of anti-SARS-CoV-2 antibodies after COVID-19 vaccination

The CORSA trial (NCT04345315) enrolled 77 hematological patients from April to June 2021 who received at least one dose of vaccine (73 patients received Comirnaty and 4 received Spikevax). Patients’ characteristics are reported in **Table 1**. Patients were stratified according to their antibody titers as described in the materials and methods section. However, due to the presence of only one patient within the 12–15 titer range, this individual was grouped with patients exhibiting titers < 12 for the purpose of analysis. This decision was made to ensure statistical consistency and avoid bias due to the underrepresentation of this subgroup.

**Table 1:** Patients’ characteristics.

Characteristic	Overall n= 77 (%)
Age at first vaccine dose	
Median (range)	71 (39-95)
Gender	
Male	39 (50.6)
Female	38 (49.4)
Disease	
B-NHL	50 (64.9)
CLL	18 (23.4)
MM	9 (11.7)
Disease status at study entry	
New diagnosis	7 (9.1)
Complete remission	30 (38.9)
Partial remission	36 (46.8)
Stable disease	2 (2.6)
Progressive disease	2 (2.6)
Median number of cycles of therapy (range)	
BTK inhibitor	20.5 (2-65)
IMiDs	–
Rituximab	6 (1-8)
Time from therapy in months, median (range)	2.0 (0.4-4.8)
Patients with maintenance therapy	14 (18.2)
Maintenance therapy	
Ibrutinib	1 (1.3)
Lenalidomide	2 (2.6)
Rituximab	11 (13.0)
Patients with immunosuppressive drugs	7 (9.1)
Patients with autoimmune disease*	11 (13.9)
Patients with allergy	12 (15.6)
Drugs	6 (7.8)
Food	3 (3.9)
Environment	2 (2.6)
Other	1 (1.3)
Previous flu vaccine	16 (20.8)
Previous diagnosis of COVID-19	2 (2.5)
AB titer - baseline value	
<15	74 (97.4)
≥15	2 (2.6)
Unknown	1

B-NHL, B-cell Non-Hodgkin Lymphoma; CLL, Chronic Lymphocytic Leukemia; MM, Multiple Myeloma; BTK, Bruton Tyrosine Kinase; IMiDs, Immunomodulatory Drugs. *Asthma, celiac disease, autoimmune thyroiditis, connectivitis, IgG deficit, hypothyroidism, psoriasis, spondyloarthritis.

Baseline peripheral blood samples were collected from 76 out of 77 patients and tested for the presence of antibodies against SARS-CoV-2 Spike S1/S2 protein. Seventy-four out of 76 patients (97%) had negative serology before vaccination while two were positive. For the latter two, one had a previous diagnosis of COVID-19, while the second did not. For 75 patients, analysis of antibody response after the first dose of vaccine was performed. Two patients were evaluated only after the first vaccine dose as one patient died before the second dose due to disease progression and one was lost to follow-up after the first dose. Two patients were evaluated only after the second dose (no sample collection was performed after the first dose).

**Figure 1:**
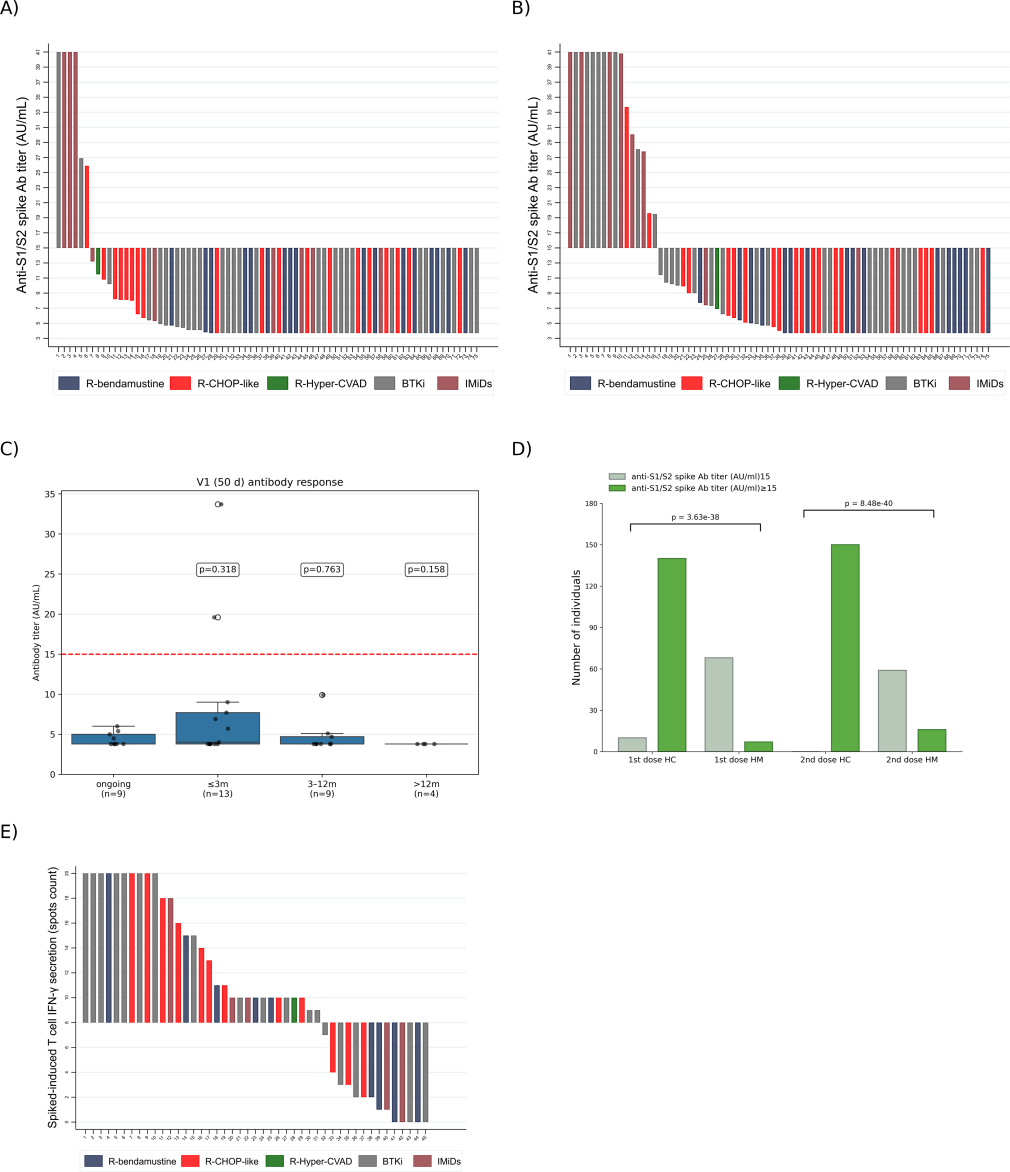
Vaccine-induced immune responses in HM patients under treatment and Healthy Controls (HC). **(A)** Anti-S IgG titer for each individual under personal anti-cancer treatment is depicted after one vaccine dose. **(B)** Anti-S IgG titer for each individual under personal anti-cancer treatment is depicted after the second vaccine dose. **(C)** Antibody response at V1 (50 days) stratified by time since rituximab treatment. **(D)** Comparison of Anti-S IgG titer in patients with HM and healthy controls (HC) after each vaccine dose. **(E)** Ex vivo Spike protein-induced T cell IFN-γ secretion for 45 individual patients under treatment (color legend) that received two vaccine doses. R-bendamustine, rituximab in combination with bendamustine; R-CHOP-like, rituximab-based combination regimens including cyclophosphamide, doxorubicin, vincristine, and prednisone (or equivalent regimens); R-Hyper-CVAD, rituximab combined with hyperfractionated cyclophosphamide, vincristine, doxorubicin, and dexamethasone; BTKi, Bruton’s tyrosine kinase inhibitors; IMiDs, immunomodulatory drugs.

After the first vaccine dose, 69 patients (92.0%) did not show any humoral response. Most non-responders were receiving rituximab-based or BTKi-containing regimens (**Supplementary Table 1**, **Figure 1A**). Among patients who were no longer in treatment but had received rituximab in the 6 months before vaccination, the median time from the end of treatment (EOT) was 2.8 months (range 1.3–5.5 months), and no difference was associated with gender. Only 6 patients (8.0%) showed an antibody response after the first dose.

After the second dose, 59 out of 75 patients (78.7%) did not show any humoral response. Lymphocyte count was deemed not informative because of overlapping ranges and because no test to separate pathological from non-pathological lymphocytes was performed. Among patients who did not respond after the complete vaccination cycle, the majority were receiving rituximab-based regimens, which were associated with the lowest response rate (5.4%), followed by BTKi (27.6%) and IMiDs (66.7%) (**Supplementary Table 2**, **Figure 1B**).

One of the two patients with previous SARS-CoV-2 infection did not develop post-vaccination antibodies (anti-S1/S2 antibodies level after second dose= 11.4 AU/mL; no baseline level available); the second one had positive anti-S antibodies at baseline (24 AU/mL) that remained stable after the first (25 AU/mL) and the second vaccine dose (19 AU/mL).

To investigate whether the timing of rituximab discontinuation relative to vaccination influences the humoral response, we stratified patients according to the interval between treatment cessation and the first vaccine dose. At V1 (50 days post-vaccination), no statistically significant differences in antibody titers were observed between patients receiving ongoing rituximab treatment and those who had discontinued therapy at different time intervals prior to vaccination. Pairwise comparisons of Spike-specific antibody titers showed no significant differences between patients who had stopped rituximab within 3 months (p = 0.318), 3–12 months (p = 0.763), or over 12 months (p = 0.158) and patients who were under Rituximab treatment (**Figure 1C**).

We compared the antibody response of patients to the reference population of 150 sex-matched (1:2) HC working at the Cancer Center where patients were enrolled and who received the BTN162b2 vaccine from January to March 2021, according to the Italian guidelines. Three of these subjects had a confirmed diagnosis of COVID-19 before vaccination. After the first vaccine dose, 140 out of 150 (93%) subjects had antibody levels above the threshold compared to only 9% of the hematological patients cohort. After the second dose, 100% of HC had a sustained humoral response compared to 21% only for the enrolled patients (**Table 2**, **Figure 1D**). The difference in seroconversion rates between HC and HM was highly significant at both timepoints (Chi-square test, p < 0.0001 after both the first and second dose), confirming a strong association between disease status and impaired humoral response. It should be noted that the median age of the reference HC population was significantly lower than the patients’ age (50 vs 71 median age).

**Table 2:** Comparison of antibody response in patients and health care professionals.

	Ab titer<15	Ab titer≥15	Total	Proportion of subject >15 (95%CI)
After the first dose				
HM subgroup	68	7	75	9.3% (3.8%-18.3%)
HC subgroup	10	140	150	93.3% (88.1%-96.8%)
After the second dose				
HM subgroup	59	16	75	21.3% (12.7%-32.3%)
HC subgroup	0	150	150	100.0% (97.5%-100.0%)

Ab, Antibody; HM, Hematological Malignancies; HC, Healthy Controls.

### COVID-19 vaccines induce a sustained anti-viral T cell response in hematological patients

To assess vaccine-induced SARS-CoV-2-specific T cell responses, the T-SPOT.COVID assay was performed in 49 of 77 patients with hematological malignancies; four patients with indeterminate results were excluded from the analysis. Out of the remaining 45, 31 (68.9%) were positive after being exposed to the S antigen, but not to the N antigen (**Table 3**), while 14 (31.1%) patients did not show any T cell response. This proves a specific anti-Spike response after vaccination. Interestingly, in one case (2.1%) with a known history of SARS-CoV-2 infection, we observed positivity for both the S and the N antigen, suggesting that both panels were sufficiently sensitive to detect previous exposures to SARS-CoV-2 proteins.

**Table 3:** Relation between patients’ humoral and T cell responses (T-SPOT.COVID Test), expressed as Ab titers and IFN-γ spot formation against S protein (Panel A) and N protein (Panel B).

IFN-γ spots	Ab titer<15 n=45 (%)	Ab titer≥15 n=4 (%)	Total n=49 (%)	P value (McNemar)
S protein				
Neg <8	12 (29.3)	2 (50.0)	14 (31.1)	<0.001
Pos ≥8	29 (70.7)	2 (50.0)	31 (68.9)	
Undetermined	4	0	4	
N protein				
Neg <8	41 (100.0)	3 (75.0)	44 (97.8)	0.250
Pos ≥8	0 (0.0)	1 (25.0)	1 (2.2)	
Undetermined	4	0	4	

Ab, Antibody, Neg, Negative; Pos, Positive.

Interestingly, 69% of the patients with negative serology after both vaccine doses had a T cell response (**Figure 1E**).

### LP showed defective spike-specific clonal breadth and clonal depth after vaccination

**Figure 2:**
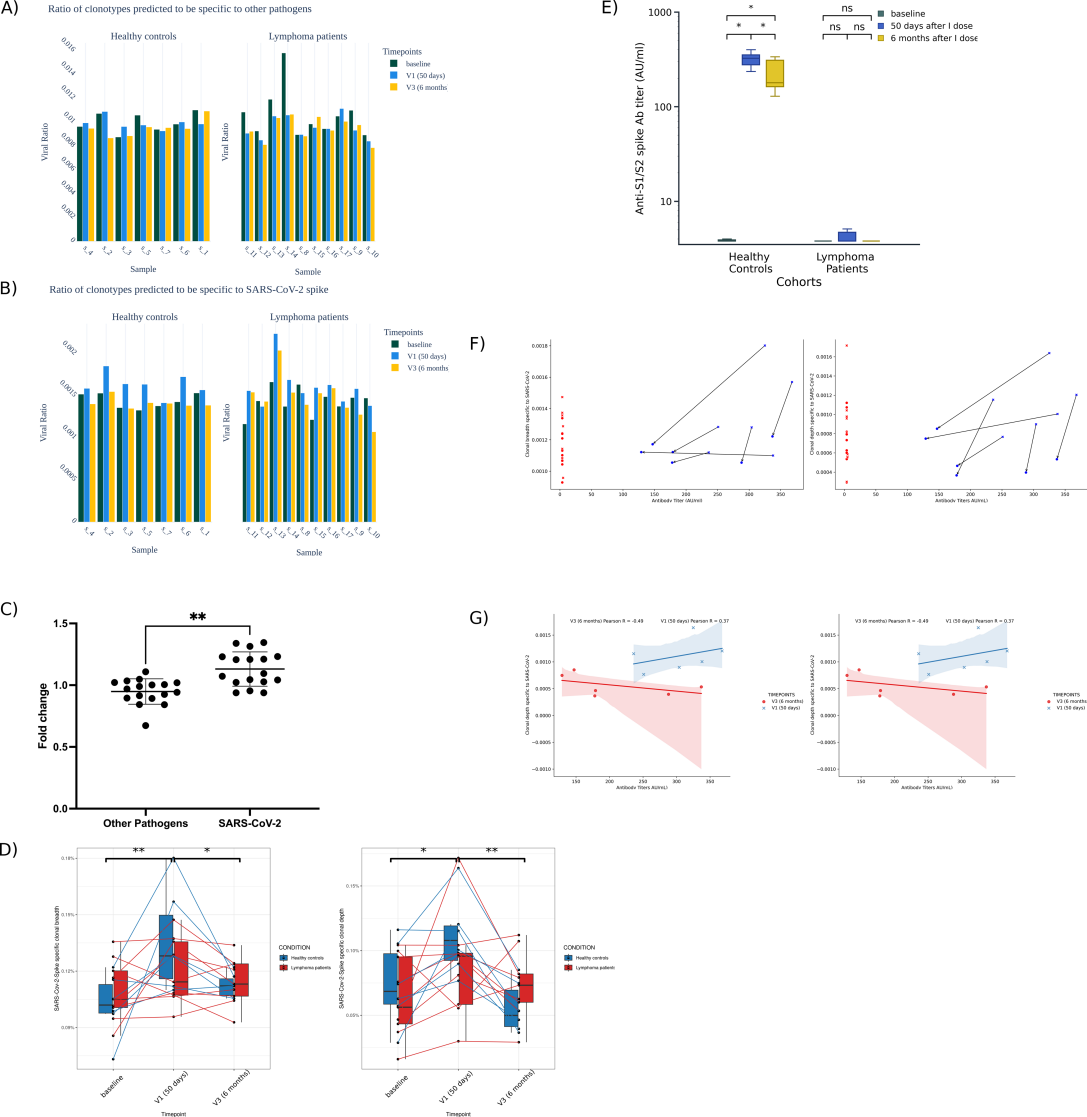
In depth analysis of Spike-specific immune responses in lymphoma patients (LP) and healthy controls (HC). The viral ratio of unique TCR clonotypes of HC and LP before (baseline) and after each vaccine dose (V1 and V3) that are predicted to recognize **(A)** pathogens epitopes different from SARS-CoV-2 and **(B)** SARS-CoV-2-Spike epitopes. **(C)** Fold changes of viral ratios calculated at V1/viral ratios at baseline for TCRs associated with other pathogens and with SARS-CoV-2-Spike. **(D)** SARS-CoV-2-Spike specific clonal breadth (diversity) and clonal depth (expansion) throughout the vaccination schedule. **(E)** Assessment of Anti-S IgG titer at baseline, 50 days and 6 months after vaccination in HC and LP cohorts. Paired comparisons of antibody titers across timepoints (baseline, V1, and V3) were performed separately within each cohort using the Wilcoxon signed-rank test. p-values were adjusted for multiple comparisons using the Holm–Bonferroni method. All tests were two-sided, ns = not significant. **(F)** SARS-CoV-2-Spike specific clonal breadth and clonal depth and corresponding antibody titers measured in HC and LP at 50 days and 6 months post-vaccination. **(G)** Correlation between humoral and cellular immune responses in HC. At 50 days post-vaccination, anti-Spike IgG levels positively correlate with T cell response breadth (R = 0.49) and depth (R = 0.37). At 6 months, this shifts to low correlation (breadth: R = 0.24; depth: R = –0.49). ns P > 0.05, *P ≤ 0.05, **P ≤ 0.01, ***P ≤ 0.001, ****P ≤ 0.0001.

The characteristics of LP and HC that were selected for TCR sequencing are shown in **Supplementary Table 3**. To test if TCR frequencies were specifically associated with the recognition of the SARS-CoV-2-Spike and not due to a general effect of the vaccine, we analyzed the TCR repertoire referred to the recognition of different pathogens. Other pathogens and SARS-CoV-2-Spike specific viral ratios are shown in **Figures 2A, B** and **Supplementary Tables 4**, **5**. The analysis revealed a statistically significant difference (p = 0.0012), with a greater increase observed in the SARS-CoV-2-Spike condition (mean = 1.131) compared to other pathogens (mean = 0.949) among vaccinated individuals. This indicates that the vaccine is able to skew the TCR repertoire towards SARS-CoV-2 spike specificity at 50 days from the administration of the first vaccine dose (**Figure 2C**, **Supplementary Table 4**).

Clonal breadth was defined as the proportion of SARS-CoV-2-specific TCRs among total unique TCR sequences and evaluated at baseline, V1, and V3 in LP and HC individuals using a Linear Mixed Model. A significant increase in clonal breadth was observed from baseline to V1 in HC (p = 0.0013), followed by a decline at V3 (p = 0.0265). Conversely, LP samples did not exhibit a statistically significant change over the same period, with clonal breadth remaining relatively stable between V1 and V3. Furthermore, the interaction term for group and time (HC vs. LP at V1) was negative and significant (p = 0.0407), indicating that LP had a significantly impaired diversification of response compared to HC at 50 days. No significant changes were detected in either group at V3 (**Figure 2D**, left). SARS-CoV-2-specific clonal depth showed significant temporal dynamics in HC, with a marked increase from baseline to V1 (p = 0.0389), followed by a reduction at V3 (p = 0.0018), both statistically significant. In contrast, no relevant changes were observed in clonal depth across time points in LP.

No significant difference between HC and LP was detected at V1 and V3.

### Impaired antibody response to SARS-CoV-2 vaccination in LP compared to HC

To illustrate the differences in vaccine-induced antibody responses, **Figure 2E** compares the trajectory of SARS-CoV-2 antibody titers over time between LP and HC following vaccination. In the HC, antibody titers significantly increased 50 days after vaccine administration, showing the highest titers and followed by a decline after six months (p < 0.05). In contrast, LP exhibited consistently low antibody titers across all time points, with no significant differences observed between baseline, V1, and V3. To assess how this humoral and the adaptive T cell responses are related to each other, clonal breadth and clonal depth were correlated with the antibody titer at each time point. In the absence of significant antibody titers for most LPs, there is no correlation with T cell response, confirming an overall impaired immune response (**Figure 2F**). For the HC cohort, a positive correlation between humoral and Spike-specific T cell response diversification (Pearson R = 0.49; **Figure 2G**) and expansion (Pearson R = 0.37; **Figure 2G**) can be appreciated at 50 days after the first dose of vaccine. These data suggest a coordinated immune response in healthy individuals at early stages of vaccination. However, the scenario at 6 months post-vaccination changes completely. At this time, higher antibody titers inversely correlate with SARS-CoV-2 specific T cell response breadth (Pearson R = 0.24; **Figure 2F**) and depth (Pearson R = –0.49; **Figure 2F**).

### Vaccination-driven convergence and subsequent divergence in SARS-CoV-2-Specific V gene usage among LP and HC

**Figure 3:**
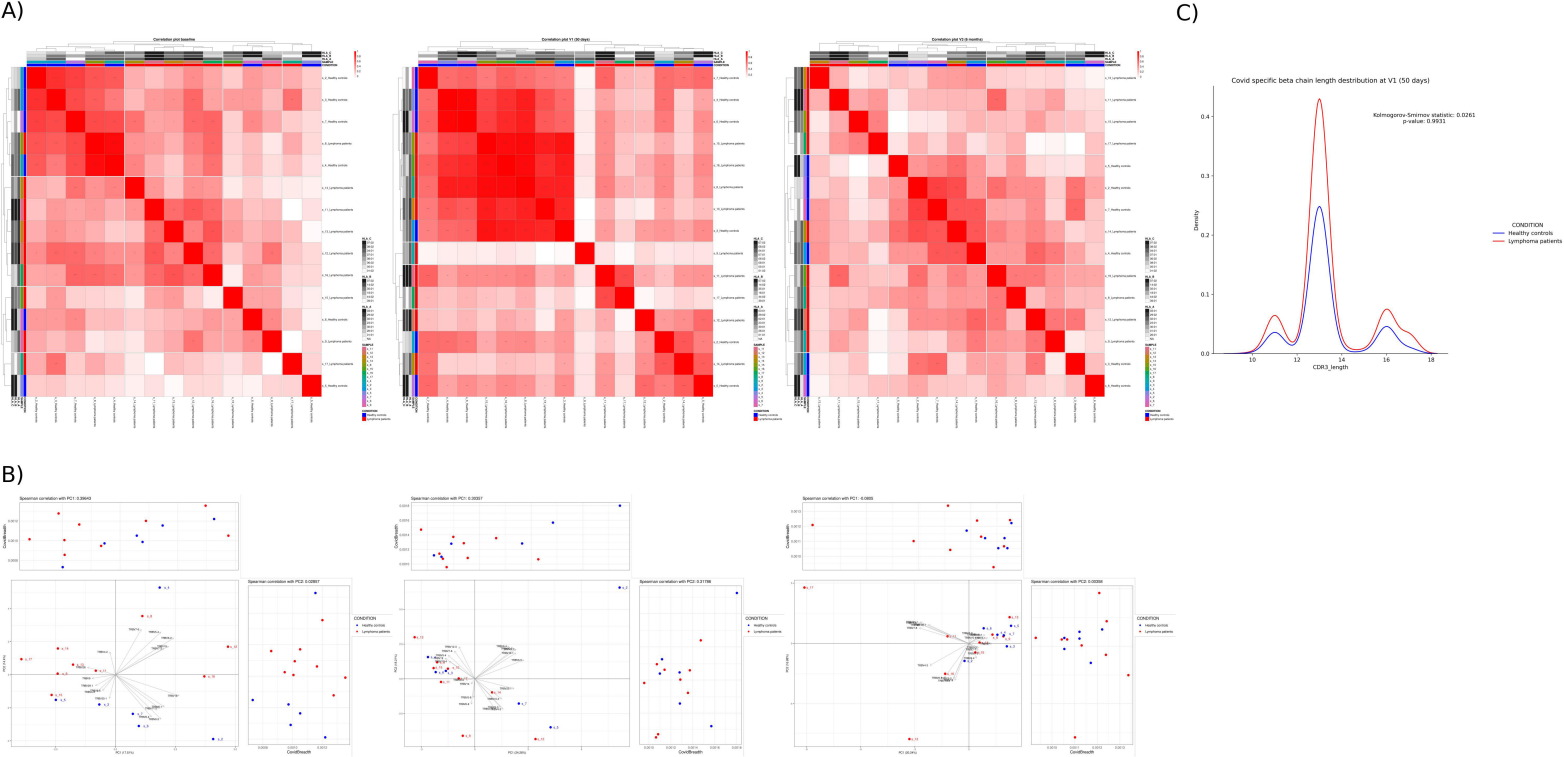
SARS-CoV-2-Specific V gene usage during VDJ rearrangement among LP and HC. **(A)** Heat map of SARS-CoV-2-specific V gene usage in HC and LP at baseline, and after vaccination (V1 and V3). Clustering analysis reveals five distinct clusters (C1–C5) based on V gene usage patterns. At baseline, HC and LP form separate clusters, converge at V1 post-vaccination, and re-segregate into multiple clusters by V3, indicating dynamic shifts in the TCR repertoire over time in response to vaccination. **(B)** PCA of SARS-CoV-2-specific V gene usage and clonal breadth in healthy controls HC and LP at baseline, V1, and V3. PCA plots show sample distribution across timepoints, with clear separation between HC and LP at baseline, primarily along PC2, associated with HLA-A and HLA-B gene usage. This separation diminishes after vaccination (V1 and V3), suggesting convergence of TCR repertoire features. Right and upper panels indicate the contribution of specific Spike-associated V genes to PC2 and PC1, respectively. **(C)** Analysis of CDR3 length at V1 revealed no significant differences between HC and LP, with both groups showing an average length of approximately 13 amino acids.

We investigated whether there was any difference in the utilization of the specific SARS-CoV-2 V genes between the two cohorts and whether this changed at different time points, as a consequence of vaccination. For each time point, we correlated the samples for V gene usage among the entire TCR repertoire to see if any V segments emerged as preferentially used during the VDJ rearrangement, but we did not observe any statistically different results (**Supplementary Figure 2**). We performed the same analysis but correlating the subjects according to the use of V genes associated with SARS-CoV-2-specific TCRs. From this analysis depicted in **Figure 3A**, we discovered that, before vaccination (baseline), HC and LP appear to form two distinct groups (Cluster 1 and Cluster 2) based on V gene usage, while a convergence towards a unique cluster can be observed at 50 days after vaccination start (**Figure 3A**, central heatmap), indicating that vaccination is driving the immune system towards the selection of specific V gene rearrangements in both cohorts. Importantly, the V gene usage repertoire diverged again at 6 months after vaccination start and LP and HC again appear mostly as separated clusters (Cluster 3, Cluster 4, Cluster 5) as the immune response fades with time in a way resuming their original differences (**Figure 3A**). However, to assess the similarity of the overall cluster assignments, the Adjusted Rand Index (ARI) was computed between the baseline and the 6-months clustering. An ARI score of 0.589 highlighted a moderate though not perfect similarity. While some samples belonged to the same cluster, a large portion of the cohort saw a change in the cluster membership.

Principal component analysis (PCA) was used to identify the main factors responsible for sample variability in terms of SARS-CoV-2-specific V gene usage. Clonal breadth was also added to PCA to assess how this dimension related to PC1 and PC2, respectively (**Figure 3B**). Interestingly, HC and LP samples were best separated on HLA-A and HLA-B (PC2 dimension) while clonal breadth did not segregate well the two cohorts at baseline (**Figure 3B**). Importantly, this distinction completely disappeared following vaccination, both at 50 days and at 6 months, as shown by the mixed distribution between HC and LP samples in the central and right panel of **Figure 3B**, respectively. This suggests that vaccination imposes high selective pressure on the type of V genes used for Spike-specific TCRs (**Figure 3B**). For a detailed description of PCA variance components, see **Supplementary Figure 3**.

Following pre-processing and sequence alignment, the length of each TCRβ CDR3 sequence was determined and analyzed to see if we could detect associations between the length of CDR3 associated with the response to vaccine-derived antigens and the two cohorts at 50 days, where the convergence of V gene usage was highlighted by the previous analysis. In addition, we did not observe significant differences in CDR3 length (13 amino acids in average) between HC and LP (**Figure 3C**).

Stratification based on individual HLA expression was not significant (**Supplementary Table 5**). Concordance between the two HLA prediction tools hla3 and HLAGuessr was 68.6%, with 35 out of 51 predictions matching across the three HLA loci assessed for each of the 17 samples.

## Discussion

In this study, we first investigated the humoral and cellular responses to two doses of mRNA COVID-19 vaccines (Comirnaty or Spikevax) in patients with hematological malignancies, selected among those receiving lymphocyte-directed therapies, as this population is at increased risk of mounting suboptimal responses to vaccination.

Our results showed an overall reduced vaccine immunogenicity in HM patients under treatment. In particular, the humoral response was nearly abolished in patients receiving a BTKi or rituximab-based regimen, with only 28.6% and 5.4% of them with a measurable anti-S Ab titer after the second dose, respectively. BTKi have been demonstrated to impact the humoral response to vaccines, possibly due to their inhibitory action against the BCR pathway (22, 23, 34). These data for HM patients are in line with the Comirnaty vaccine’s reported efficacy in CLL patients (35) and with other studies, highlighting the importance of booster doses (16, 36, 37). In the absence of antibody neutralization data, the antibody titer and therapeutic protection cannot be directly linked. Similar low humoral response rates have also been described after vaccination against different pathogens, including after pneumococcal conjugated (PCV13) and polysaccharide vaccine (PPSV23), HepB-CpG vaccine, and Influenza A and B vaccine in CLL patients (38–40) and after Varicella zoster (VZV) vaccine, H1N1 vaccine, Streptococcus pneumoniae polysaccharide vaccine and Hemophilus influenzae type B (Hib) conjugate vaccine in patients receiving an anti-CD20 treatment (20, 21, 41). Although sterilizing immunity can be obtained only through high titers of neutralizing antibodies (42), B cells do not appear to be strictly necessary to recover from COVID-19. This is evidenced by the successful recovery of people suffering from X-linked agammaglobulinemia (43) and by studies reporting that the majority of patients with multiple sclerosis undergoing anti-CD20 immunotherapy recover from COVID-19 and do not suffer from an increased risk of death (44), thus supporting the presence of compensatory immune response pathways.

IFN-γ release assay and flow cytometry-based analyses have been used extensively to estimate T-cell responses against SARS-CoV-2. Grifoni et al. identified a Th1 immune response, as substantial interferon IFN-γ was produced, in the majority of convalescent patients, suggesting its importance in contrasting progression and resolution of disease (45). A study by Kuppalli et al. identified a suppressed cellular immune response as one of the hallmarks of severe COVID-19 patients (46), and Toor et al. further assessed the importance of T cell subpopulations in regulating the response to, and the clinical course of, the disease (47). Thus, we analyzed the T cell response by measuring IFN-γ production after exposure to the S and N viral antigens. Contrary to what we observed for the humoral response, we found that more than two-thirds of our patient population showed a positive response to the S antigen, even in those who did not develop any measurable Ab titer after a full vaccination course. Specifically, 69% of patients with negative serology after both vaccine doses had a T cell response, in line with what other studies have reported. Indeed, Marasco et al. showed that 74% of seronegative lymphoid malignancy patients still developed a T cell response, suggesting that vaccine-specific T cell immunity is not completely hindered by B cell aplasia and may provide some level of protection in patients (17).

Although cross-reactivity to other viruses should be taken into account, the high percentage of responders is closer to that of subjects that have recovered from COVID-19 compared to unexposed people (45), suggesting that the vaccine was able to induce a T cell response. This is relevant, since a responsive T cell population could contribute to viral clearance and potentially promote a less severe clinical course despite HM patients not seroconverting after vaccination. Moreover, it suggests that measuring T cell activation in addition to Ab titers could offer a more comprehensive estimate of COVID-19 vaccine immunogenicity in people receiving immunosuppressive therapies.

In a study by Gurion et al. involving 162 LP, it was described that only 51% developed positive antibody responses after two doses of the BNT162b2 vaccine. The authors also underlined that factors such as receiving the last anti-CD20 monoclonal antibody dose within 12 months before vaccination and having active lymphoma were associated with reduced vaccine responses (48), while T cell response was not reported. Our study instead shows a non-significant difference in terms of spike-specific antibody responses after two doses of SARS-CoV-2 vaccine in patients who have interrupted the rituximab course at different times (from 3 to more than 12 months) compared to patients with ongoing rituximab treatment suggesting that suspension of rituximab for vaccination purposes won’t benefit a serological antiviral response in those patients. Similarly, the PROSECO study in the UK analyzed 457 LP and found that post-vaccination anti-SARS-CoV-2 IgG levels were significantly lower compared to HC (49), while 63% of all patients showed antigen-specific T cell responses, evaluated by ELISPOT assay, which increased following a third dose. Similarly, Liebers et al. assessed that 50% of LP without a seroconversion still showed measurable T-cell responses (50). Despite detectable T cell reactivity, it remains remarkable that LP remains a fragile and sensitive population to the severe consequences of COVID-19 (15).

Novel methods like the sequencing of TCRs can provide additional information regarding T cell repertoire architecture, including clonality and diversity. To investigate more in detail the T cell response to vaccination, we performed TCR repertoire sequencing to compare more accurately the diversity and the depth of response in two randomly selected subgroups of HC and LP. High-resolution TCR sequencing allows to quantify the clonal variability and relative expansion of clonotypes associated with viral recognition, as shown in previous work from us and others (51, 52). Our findings highlighted differences in TCR clonal breadth and depth dynamics between the two cohorts at 50 days post-vaccination. HC demonstrated a clear expansion of SARS-CoV-2-specific TCR clonotypes shortly after vaccination, as indicated by the significant increase in clonal breadth and depth at V1. This response mirrors typical vaccine-induced T cell activation and expansion, followed by contraction at later time points (V3) (53, 54). In contrast, LP displayed a blunted T cell response, with minimal changes across time and a significantly attenuated breadth increase at V1, indicating a reduced ability to diversify the cellular response following vaccination. This altered T cell response occurs in the context of a severely compromised humoral response, as evidenced by persistently low antibody titers across all time points. While healthy individuals developed strong antibody responses that peaked at 50 days and declined after six months, LP showed no significant antibody increase. Together, these findings indicate that immunocompromised LP do not mount immune responses comparable to those observed in healthy vaccinated individuals, particularly in terms of response dynamics and coordination.

Notably, Papalexandri et al. reported significant skewing of the TCRβ gene repertoire in patients receiving Rituximab post-allogeneic hematopoietic cell transplantation, often accompanied by oligoclonal expansions of CD8^+^ T cells with large granular lymphocyte (T-LGL) phenotypes (55). Their data suggest that Rituximab, may indirectly perturb T cell homeostasis, possibly through altered immune interactions. This observation is consistent with the reduced clonal dynamics and limited repertoire diversification in LP post-vaccination in our study. Despite this, V gene usage analysis revealed patterns in antigen-specific repertoire architecture. While global V gene usage did not differ significantly between the cohorts, clustering based on SARS-CoV-2-specific V gene usage showed distinct groupings at baseline, followed by a convergence at V1 (50 days post vaccination), and a subsequent divergence at V3. This convergence may reflect shared vaccine-induced responses, although further validation is needed. PCA analysis, similarly, showed a baseline separation between HC and LP that was reduced after vaccination, suggesting that vaccination induced partially overlapping T cell responses across individuals. However, these patterns should be interpreted cautiously given the limited sample size.

The convergence trend between the two repertoires after vaccination may align with other works reporting T cell response in larger populations of LP, evaluated in terms of IFN-γ release (50). T cell responses were assessed in samples collected at a median of 17 days after the second vaccination dose, which can be considered to be similar to our V1. Among the vaccinated LP, 58% exhibited activation of T cells after incubation with 2 overlapping peptide pools representing the complete spike protein. This percentage rises to 70% in HC. This result suggests that a certain degree of Spike-specific response is indeed present also in LPs.

Greenberger et al. comprehensively evaluated both humoral and cellular immune responses to SARS-CoV-2 mRNA vaccination in a large cohort of patients with hematological malignancies, predominantly those with B-cell neoplasms (16). Their findings highlighted a dissociation between antibody and T cell responses, in line with our findings. While a proportion of patients failed to develop antibodies, many still exhibited T cell responses. Importantly, they observed a greater breadth of Spike-associated T cell responses in seropositive patients compared with seronegative patients and a trend for increased breadth of Spike-specific T cells in response to vaccination with mRNA-1273 compared with the BNT162b2 vaccine. Our study extends those observations by incorporating TCR analysis, showing that seronegative LP exhibit reduced clonal breadth compared to HC at early timepoints Albeit the relatively small sample sizes, these findings are consistent with previously reported trends. Since LP were not followed up for COVID-19 symptoms, a direct correlation between immune responses and clinical protection cannot be clearly established.

To further delineate how different therapies modulate immune responses to vaccination, future studies should aim for larger cohorts and include detailed analysis of treatment regimens. In addition, longer follow-up will be necessary to evaluate the durability and potential clinical relevance of these immune responses even after full vaccination schedule. Another limitation of the study is the limited sample size, particularly for subgroup analyses by diagnosis and treatment and for the TCR sequencing subset. Therefore, these findings should be considered exploratory and require further confirmation in larger cohorts.

Taken together, our methodologies and findings highlight measurable differences in vaccine-induced immune responses between healthy individuals and LP, supporting the use of TCR repertoire analysis to further characterize cellular immune responses. TCR repertoire profiling provides complementary information to conventional assays by describing the clonal architecture of the T cell response, particularly in contexts where humoral immunity is compromised. As a valuable tool to assess vaccine immunogenicity beyond cytokine secretion and antibody titers, particularly in contexts where humoral immunity is compromised. Our study provides a detailed characterization of the immune alterations associated with vaccination in patients bearing hematological malignancies and may inform future studies aimed at optimizing vaccination strategies in immunocompromised individuals. To the best of our knowledge, the CORSA trial is the first study assessing the effects of the COVID-19 vaccine and the relationship between serological and T cell responses at the TCR sequence level in a unique Italian population of LP and HC. Our study further supports the recommendation to also vaccinate patients undergoing severely immunosuppressive treatments with mRNA COVID-19 vaccine, as even a partial immune response may be clinically relevant and contribute to reducing disease severity and that suspension of rituximab treatment for vaccination in LPs is surely not justified by the observed immunological responses.

## Data Availability

The datasets presented in this study can be found in online repositories. The names of the repository/repositories and accession number(s) can be found below: https://ega-archive.org/datasets/EGAD50000001714, EGAD50000001714.
