# Supplementary material for: CORSA study finds spike-specific blunted immune responses in lymphoma patients after SARS-CoV-2 vaccine
Source: Front Immunol. 2026 May 20;17:1756325. doi: 10.3389/fimmu.2026.1756325 (PMC13230124; doi:10.3389/fimmu.2026.1756325)
Supplement: Supplementary file 1 [file Table1.docx]

**Supplementary materials**

**Supplementary Table 1.** Baseline characteristics of patients stratified by Ab titer after the first dose of SARS-CoV-2 vaccine.

|  | **Ab titer<15**  **n=69 (%)** | **Ab titer≥15**  **n=6 (%)** | **Total**  **n=75** |
| --- | --- | --- | --- |
|  |  |  |  |
| Age at first vaccine dose,   Median (range) | 71.4 (38.9-94.9) | 56.3 (44.7-77.3) | 71.1 (38.9-94.9) |
| Gender |  |  |  |
| Male | 36 (52.2) | 2 (33.3) | 38 (50.7) |
| Female | 33 (47.8) | 4 (66.7) | 37 (49.3) |
|  |  |  |  |
| Disease |  |  |  |
| B-NHL | 47 (68.1) | 1 (16.7) | 48 (64.0) |
| CLL | 16 (23.2) | 2 (33.3) | 18 (24.0) |
| MM | 6 (8.7) | 3 (50.0) | 9 (12.0) |
|  |  |  |  |
| Disease status at study entry |  |  |  |
| New diagnosis | 6 (8.7) | 1 (16.7) | 7 (9.3) |
| Complete remission | 29 (42.0) | 0 (0.0) | 29 (38.7) |
| Partial remission | 31 (44.9) | 4 (66.7) | 35 (46.6) |
| Stable disease | 2 (2.9) | 0 (0.0) | 2 (2.7) |
| Progressive disease | 1 (1.5) | 1 (16.7) | 2 (2.7) |
|  |  |  |  |
| Therapy for hematologic disease |  |  |  |
| BTK inhibitor | 27 (39.1) | 2 (33.3) | 29 (38.7) |
| IMiDs | 6 (8.7) | 3 (50.0) | 9 (12.0) |
| Rituximab regimen | 36 (52.2) | 1 (16.7) | 37 (49.3) |
|  |  |  |  |
| N cycle of therapy, median (range) | 6 (1-65) | 6 (1-27) | 6 (1-65) |
|  |  |  |  |
| Ongoing therapy | 40 (58.0) | 4 (66.7) | 44 (58.7) |
|  |  |  |  |
| Time from therapy in months, median (range) | 2.0 (0.5-4.8) | 0.7 (0.7-0.7) | 1.9 (0.5-4.8) |
|  |  |  |  |
| Immunosuppressive drugs | 7 (10.1) | 0 (0.0) | 7 (9.3) |
|  |  |  |  |
| Autoimmune disease | 11 (15.9) | 0 (0.0) | 11 (14.7) |
|  |  |  |  |
| Presence of allergy | 10 (14.5) | 2 (33.3) | 12 (16.0) |
|  |  |  |  |
| Flu vaccine | 13 (18.8) | 3 (33.3) | 15 (20.0) |
|  |  |  |  |
| Previous diagnosis of COVID-19 | 0 (0.0) | 1 (16.7) | 1 (1.3) |
|  |  |  |  |
| Baseline WBC, median (range) | 5.15 (1.33-40.89) | 6.99 (2.83-13.28) | 5.15 (1.33-40.89) |
| Baseline Neu, median (range) | 2.99 (0.11-28.39) | 4.12 (1.05-8.34) | 2.99 (0.11-28.39) |
| Baseline Lymp, median (range) | 1.13 (0.20-33.64) | 1.64 (0.55-6.52) | 1.16 (0.20-33.64) |

*WBC = white blood cells; Neu = neutrophil; Lymp = lymphocytes; ns = not significant.

**Supplementary Table 2.** Baseline characteristics of patients stratified by Ab titer after the second dose of the SARS-CoV-2 vaccine.

|  | **Ab titer<15**  **n=59 (%)** | **Ab titer≥15**  **n=16 (%)** | **Overall**  **n=75 (%)** | **p-value** |
| --- | --- | --- | --- | --- |
|  |  |  |  |  |
| Age at first vaccine dose,  Median (range) | 71.3 (38.9-94.9) | 64.9 (44.7-79.6) | 71.1 (38.9-94.9) | ns |
|  |  |  |  |  |
| Gender |  |  |  |  |
| Male | 31 (52.5) | 7 (43.8) | 38 (50.7) | ns |
| Female | 28 (47.5) | 9 (56.2) | 37 (49.3) |  |
|  |  |  |  |  |
| Disease |  |  |  |  |
| B-NHL | 45 (76.3) | 4 (25.0) | 49 (65.3) | <0.001 |
| CLL | 11 (18.6) | 6 (37.5) | 17 (22.7) |  |
| MM | 3 (5.1) | 6 (37.5) | 9 (12.0) |  |
|  |  |  |  |  |
| Disease status at study entry |  |  |  |  |
| New diagnosis | 6 (10.2) | 1 (6.2) | 7 (9.3) | ns |
| Complete remission | 25 (42.3) | 3 (18.7) | 28 (37.3) |  |
| Partial remission | 26 (44.1) | 10 (62.5) | 36 (48.0) |  |
| Stable disease | 1 (1.7) | 1 (6.3) | 2 (2.7) |  |
| Progressive disease | 1 (1.7) | 1 (6.3) | 2 (2.7) |  |
|  |  |  |  |  |
| Therapy for hematologic disease |  |  |  |  |
| BTK inhibitor | 21 (35.6) | 8 (50.0) | 29 (38.7) | <0.001 |
| IMiDs | 3 (5.1) | 6 (37.5) | 9 (12.0) |  |
| Rituximab regimen | 35 (59.3) | 2 (12.5) | 37 (49.3) |  |
|  |  |  |  |  |
| N cycle of therapy, median (range) | 6 (1-65) | 11 (1-49) | 6 (1-65) |  |
| Unknown | 0 | 1 | 1 |  |
|  |  |  |  |  |
| Ongoing therapy | 32 (54.2) | 12 (75.0) | 44 (58.7) | ns |
|  |  |  |  |  |
| Time from therapy in months, Median (range) | 2.8 (1.3-5.5) | - | 2.7 (1.2-5.5) | - |
|  |  |  |  |  |
| Immunosuppressive drugs | 5 (8.5) | 2 (12.5) | 7 (9.3) | ns |
|  |  |  |  |  |
| Autoimmune disease | 9 (15.3) | 2 (12.5) | 11 (14.7) | ns |
|  |  |  |  |  |
| Presence of allergy | 9 (15.3) | 2 (12.5) | 11 (14.7) | ns |
|  |  |  |  |  |
| Flu vaccine | 9 (15.2) | 6 (37.5) | 15 (20.0) | ns |
|  |  |  |  |  |
| Previous diagnosis of COVID-19 | 1 (1.7) | 1 (6.2) | 2 (2.7) | ns |
|  |  |  |  |  |
| Baseline WBC, median (range)* | 4.8 (1.3-40.9) | 6.1 (2.6-13.2) | 5.1 (1.3-40.9) | 0.016 |
| Baseline Neu, median (range)* | 2.7 (0.1-28.4) | 3.6 (1.1-8.3) | 2.9 (0.1-28.4) | ns |
| Baseline Lymp, median (range)* | 1.1 (0.2-33.6) | 1.6 (0.5-6.5) | 1.1 (0.2-33.6) | 0.019 |

*WBC = white blood cells; Neu = neutrophil; Lymp = lymphocytes; ns = not significant.

**Supplementary Table 3.** LP and HC characteristics.

| **Sample Code** | **Sex** | **Age** | **Ab Titer (UA/ml)** | **Ab Titer V1 (50 days) (UA/ml)** | **Ab Titer V3 (6 months) (UA/ml)** | **Lymphoma Classification** | **Treatment** |
| --- | --- | --- | --- | --- | --- | --- | --- |
| 633 | F | 61 | <3.80 | <3.80 | <3.80 | Follicular NH | R-comp |
| 654 | M | 77 | <3.80 | 5 | <3.80 | Follicular B NH | R-benda |
| 667 | M | 56 | <3.80 | <3.80 | <3.80 | Follicular | R-benda |
| 668 | F | 76 | 8.7 | 9.9 | 10.5 | Diffuse large B cells NH | R-comp |
| 670 | F | 60 | <3.80 | <3.80 | <3.80 | Follicular B NH | Duvelisib |
| 674 | F | 68 | <3.80 | <3.80 | <3.80 | Follicular | Rituximab |
| 676 | F | 80 | <3.80 | 5.1 | <3.80 | Marginal | Rituximab |
| 678 | F | 55 | <3.80 | <3.80 | <3.80 | Marginal | R-benda |
| 683 | F | 75 | <3.80 | <3.80 | <3.80 | Diffuse large B cells NH | R-comp |
| 687 | F | 74 | <3.80 | <3.80 | <3.80 | Follicular B NH | R-benda |
| 39465 | M | 36 | <3.80 | 236 | 178 | Healthy control | – |
| 39498 | F | 37 | <3.80 | 325 | 147 | Healthy control | – |
| 39521 | F | 31 | <3.80 | 304 | 288 | Healthy control | – |
| 39617 | F | 55 | <3.80 | 368 | 337 | Healthy control | – |
| 39678 | F | 40 | 4.0 | 338 | 129 | Healthy control | – |
| 39670 | F | 39 | <3.80 | 251 | 179 | Healthy control | – |
| 39700 | F | 46 | 60.1 | >400 | 330 | Healthy control | – |

**Note.** “Ab Titer” = Antibody titer at baseline. “V1” = 50 days; “V3” = 6 months. "<3.80" indicates values below detection thresholds. “NH” = Non-Hodgkin; “R-comp” = Rituximab + chemotherapy; “R-benda” = Rituximab + bendamustine.

**Supplementary Table 4.** Fold changes calculated as V1/baseline for other pathogens and SARS-CoV-2-Spike.

| **Fold change other pathogens** | **Fold change SARS-CoV-2 Spike** |
| --- | --- |
| 0.842 | 1.339 |
| 0.915 | 0.954 |
| 0.893 | 1.345 |
| 0.673 | 1.230 |
| 0.969 | 1.314 |
| 1.002 | 1.095 |
| 1.048 | 1.042 |
| 1.005 | 0.938 |
| 0.843 | 1.072 |
| 0.942 | 0.938 |
| 0.895 | 1.023 |
| 1.020 | 1.211 |
| 1.109 | 1.207 |
| 1.035 | 1.045 |
| 0.925 | 1.232 |
| 1.022 | 1.209 |
| 0.990 | 1.027 |

**Supplementary Table 5.** HLA typing data based on individual HLA expression inferred for each sample using the tools hla3 and GuessR, whose performance and concordance are shown here. When the boxes are empty, it means that the tool had no confidence predictions.

| **Sample** | **Locus** | **hla3**  **HLA-1** | **hla3**  **HLA-2** | **GuessR HLA-1** | **GuessR HLA-2** | **Match** |
| --- | --- | --- | --- | --- | --- | --- |
| s_1 | HLA-A | HLA-A*26:01 | HLA-A*32:01 | HLA-A*26:01 |  | HLA-A*26:01 |
| s_1 | HLA-B | HLA-B*35:01 | HLA-B*38:01 | HLA-B*35:01 | HLA-B*07:02 | HLA-B*35:01 |
| s_1 | HLA-C | HLA-C*04:01 | HLA-C*07:04 | HLA-C*04:01 | HLA-C*07:01 | HLA-C*04:01 |
| s_10 | HLA-A | HLA-A*30:01 | HLA-A*24:02 |  |  | HLA-A*30:01 |
| s_10 | HLA-B | HLA-B*35:08 | HLA-B*39:01 |  |  | HLA-B*35:08 |
| s_10 | HLA-C | HLA-C*06:02 | HLA-C*04:01 | HLA-C*06:02 | HLA-C*07:01 | HLA-C*06:02 |
| s_11 | HLA-A | HLA-A*03:01 | HLA-A*01:01 | HLA-A*03:01 |  | HLA-A*03:01 |
| s_11 | HLA-B | HLA-B*07:02 | HLA-B*08:01 | HLA-B*07:02 |  | HLA-B*07:02 |
| s_11 | HLA-C | HLA-C*07:02 | HLA-C*07:04 | HLA-C*07:02 |  | HLA-C*07:02 |
| s_12 | HLA-A | HLA-A*03:01 | HLA-A*32:01 |  |  | HLA-A*03:01 |
| s_12 | HLA-B | HLA-B*14:02 | HLA-B*51:01 | HLA-B*14:02 |  | HLA-B*14:02 |
| s_12 | HLA-C | HLA-C*08:02 | HLA-C*15:02 | HLA-C*06:02 | HLA-C*08:02 | HLA-C*08:02 |
| s_13 | HLA-A | HLA-A*29:02 | HLA-A*25:01 | HLA-A*02:01 |  | HLA-A*29:02 |
| s_13 | HLA-B | HLA-B*35:01 | HLA-B*35:03 | HLA-B*35:01 |  | HLA-B*35:01 |
| s_13 | HLA-C | HLA-C*04:01 | HLA-C*03:02 |  |  | HLA-C*04:01 |
| s_14 | HLA-A | HLA-A*02:01 | HLA-A*01:01 | HLA-A*02:01 |  | HLA-A*02:01 |
| s_14 | HLA-B | HLA-B*18:01 | HLA-B*35:08 | HLA-B*18:01 |  | HLA-B*18:01 |
| s_14 | HLA-C | HLA-C*04:01 | HLA-C*07:01 | HLA-C*07:01 |  | HLA-C*07:01 |
| s_15 | HLA-A | HLA-A*23:01 | HLA-A*29:02 | HLA-A*02:01 |  | HLA-A*23:01 |
| s_15 | HLA-B | HLA-B*35:01 | HLA-B*39:01 | HLA-B*44:03 |  | HLA-B*35:01 |
| s_15 | HLA-C | HLA-C*04:01 | HLA-C*06:02 | HLA-C*04:01 | HLA-C*07:01 | HLA-C*04:01 |
| s_16 | HLA-A | HLA-A*02:01 | HLA-A*01:01 | HLA-A*01:01 | HLA-A*02:01 | HLA-A*02:01 |
| s_16 | HLA-B | HLA-B*44:02 | HLA-B*37:01 |  |  | HLA-B*44:02 |
| s_16 | HLA-C | HLA-C*06:02 | HLA-C*07:02 | HLA-C*07:01 | HLA-C*04:01 | HLA-C*06:02 |
| s_17 | HLA-A | HLA-A*30:01 | HLA-A*31:01 |  |  | HLA-A*30:01 |
| s_17 | HLA-B | HLA-B*39:01 | HLA-B*37:01 |  |  | HLA-B*39:01 |
| s_17 | HLA-C | HLA-C*02:02 | HLA-C*06:02 | HLA-C*06:02 |  | HLA-C*06:02 |
| s_2 | HLA-A | HLA-A*26:01 | HLA-A*68:01 |  |  | HLA-A*26:01 |
| s_2 | HLA-B | HLA-B*44:02 |  | HLA-B*51:01 | HLA-B*44:02 | HLA-B*44:02 |
| s_2 | HLA-C | HLA-C*15:02 | HLA-C*05:01 | HLA-C*05:01 |  | HLA-C*05:01 |
| s_3 | HLA-A | HLA-A*02:01 | HLA-A*29:02 | HLA-A*02:01 | HLA-A*26:01 | HLA-A*02:01 |
| s_3 | HLA-B | HLA-B*35:01 | HLA-B*49:01 | HLA-B*35:01 |  | HLA-B*35:01 |
| s_3 | HLA-C | HLA-C*04:01 | HLA-C*06:02 | HLA-C*06:02 | HLA-C*07:01 | HLA-C*06:02 |
| s_4 | HLA-A | HLA-A*02:01 | HLA-A*31:01 | HLA-A*02:01 |  | HLA-A*02:01 |
| s_4 | HLA-B | HLA-B*35:01 | HLA-B*35:03 | HLA-B*35:01 | HLA-B*35:03 | HLA-B*35:01 |
| s_4 | HLA-C | HLA-C*04:01 | HLA-C*15:02 | HLA-C*07:01 |  | HLA-C*04:01 |
| s_5 | HLA-A | HLA-A*01:01 | HLA-A*02:01 | HLA-A*01:01 | HLA-A*02:01 | HLA-A*01:01 |
| s_5 | HLA-B | HLA-B*53:01 | HLA-B*07:02 | HLA-B*07:02 | HLA-B*18:01 | HLA-B*07:02 |
| s_5 | HLA-C | HLA-C*07:02 | HLA-C*17:01 | HLA-C*07:02 |  | HLA-C*07:02 |
| s_6 | HLA-A | HLA-A*26:01 | HLA-A*03:01 | HLA-A*26:01 | HLA-A*02:01 | HLA-A*26:01 |
| s_6 | HLA-B | HLA-B*07:02 | HLA-B*13:02 | HLA-B*07:02 | HLA-B*35:01 | HLA-B*07:02 |
| s_6 | HLA-C | HLA-C*07:02 | HLA-C*07:01 | HLA-C*07:02 | HLA-C*07:01 | HLA-C*07:02 |
| s_7 | HLA-A | HLA-A*24:02 |  |  |  | HLA-A*24:02 |
| s_7 | HLA-B | HLA-B*18:01 | HLA-B*55:01 | HLA-B*18:01 | HLA-B*07:02 | HLA-B*18:01 |
| s_7 | HLA-C | HLA-C*01:02 | HLA-C*07:01 |  |  | HLA-C*01:02 |
| s_8 | HLA-A | HLA-A*02:01 | HLA-A*29:02 | HLA-A*68:02 | HLA-A*02:01 | HLA-A*02:01 |
| s_8 | HLA-B | HLA-B*39:01 | HLA-B*35:01 | HLA-B*35:01 |  | HLA-B*35:01 |
| s_8 | HLA-C | HLA-C*08:01 | HLA-C*07:02 | HLA-C*07:01 | HLA-C*04:01 | HLA-C*08:01 |
| s_9 | HLA-A | HLA-A*02:01 | HLA-A*32:01 |  |  | HLA-A*02:01 |
| s_9 | HLA-B | HLA-B*18:01 | HLA-B*57:01 | HLA-B*18:01 |  | HLA-B*18:01 |
| s_9 | HLA-C | HLA-C*07:04 | HLA-C*06:02 | HLA-C*06:02 |  | HLA-C*06:02 |

**
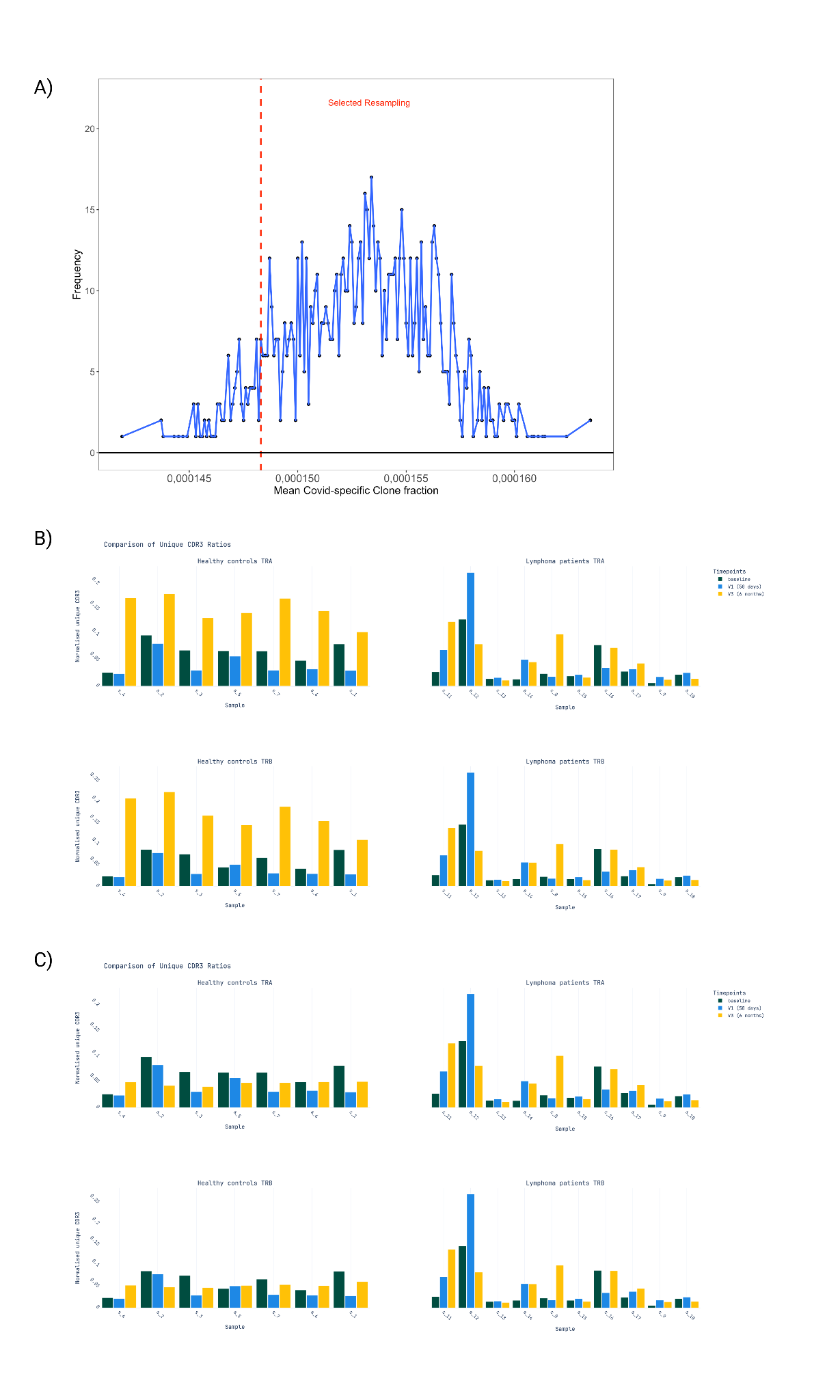
**

**Supplementary Figure 1.** Clonotypes' frequency recalculated after resampling to minimize the confounding factor of sequencing depth in V3 HC time point (A) and comparison of the repertoires before resampling (B) and after resampling (C).

**
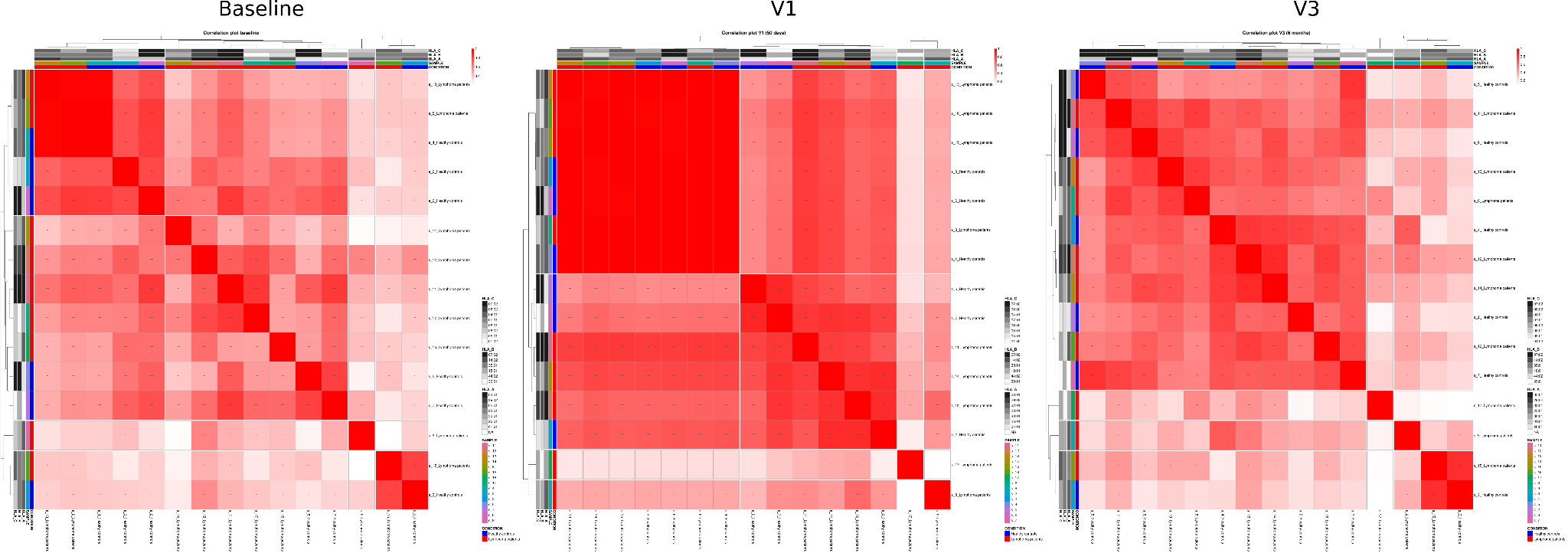
**

**Supplementary Figure 2.** V gene usage among the entire TCR repertoire for each time point, no clustering highlighted.

**
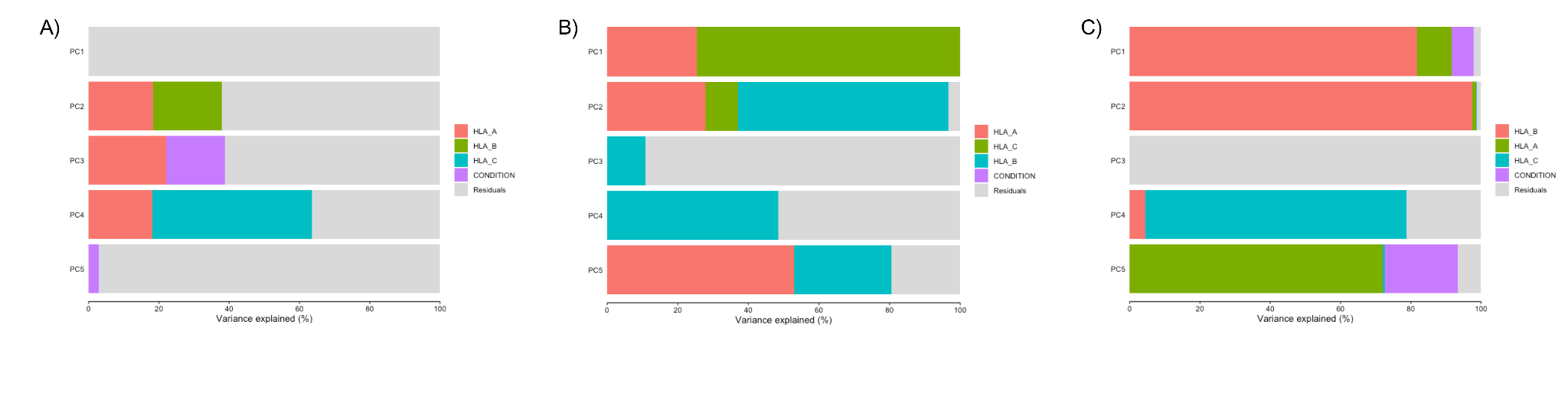
**

**Supplementary Figure 3.** SARS-CoV-2-specific V gene usage PCA variance explanation of each component, to highlight PC1 and PC2, at baseline (A), V1 (B) and V3 (C).
